# The network structure of daily stress process components: Comparing mothers of children with and without developmental disabilities

**DOI:** 10.1017/S095457942610162X

**Published:** 2026-07-07

**Authors:** Anat Zaidman-Zait, David M. Almeida, Leann Smith DaWalt, Robert S. Dembo, Jinkuk Hong, Marsha R. Mailick

**Affiliations:** 1 Department of Special Education & Educational Counseling, School of Education, Tel Aviv University, Tel Aviv, Israel; 2 Department of Human Development and Family Studies, The Pennsylvania State University, University Park, PA, USA; 3 Waisman Center, University of Wisconsin-Madison, Madison, WI, USA

**Keywords:** Caregiving, daily stress processes, depressive symptoms, developmental disabilities, mothers’, network analysis, parenting stress

## Abstract

Guided by a lifespan developmental perspective, using a network analysis approach, this study compared the structure of daily stress components in mothers of adolescents and adults with developmental disabilities (DD) and a matched sample of mothers of children without DD. We also examined whether components of daily stress were differentially associated with subsequent depression symptoms. Participants (*N* = 516; 100% female; *M* = 54.52 years, *SD* = 10.21; 94.2% White) were drawn from two cohorts: a DD cohort constructed from two linked longitudinal studies of families of adolescents and adults with autism and fragile X syndrome and a comparison group from the Midlife in United States study. Participants completed an 8-day daily telephone interview and reported depressive symptoms two years later. Findings demonstrated that the daily stress network of mothers of individuals with DD was significantly more interconnected than that of the comparison group. Stressor risk appraisal emerged as a central node in both groups, highlighting the role of cognitive appraisal in shaping stress responses. Negative affective reactivity linked daily stress components with later depressive symptoms, particularly in the DD group. Chronic caregiving stress may heighten interconnectivity within daily stress networks, reducing psychological flexibility and increasing vulnerability to daily stressors.

## Introduction

Extensive research has shown that children and adults with developmental disabilities (DD), including autism, fragile X syndrome, and intellectual disabilities, pose significant demands and challenges to their parents, who often experience elevated psychological distress and poorer physical health relative to parents of children without disabilities (Barker et al., 2014; Dembo et al., 2025; Song et al., 2018). These disparities have been largely attributed to the chronic, intensive and multifaceted stressors associated with parenting a child with DD, which require continual coping resources and ongoing adaptation (McManus et al., 2011; Seltzer et al., 2009).

In addition to primary caregiving responsibilities, the challenges associated with parenting a child with DD often proliferate, generating a cascade of secondary stressors across multiple life domains (Pearlin et al., 2005), including work, finances, marital relationships, and social connections (Bahri et al., 2023; Cidav et al., 2012; Marsack & Perry, 2018; Stabile & Allin, 2012), exacerbating parents’ vulnerability. Importantly, mothers are commonly the primary caregivers, disproportionately affected by the cumulative demands and burden of caregiving (Hsiao, 2018).

Unlike typical parenting trajectories, these responsibilities rarely diminish over time. Instead, they tend to persist, and in many cases intensify into the child’s adulthood, resulting in prolonged and often lifelong caregiving demands and sustained strain (Chamak & Bonniau, 2016; Dembo et al., 2025). However, most research has focused on parents’ stress during early childhood, with far less attention devoted to stress experiences as children with DD transition into adolescence and adulthood and as parents themselves enter midlife and older age.

Overall, the experiences of parents of individuals with DD are consistent with conceptualizations of chronic stress, defined as enduring contextual role strain that persists over extended periods and requires ongoing adaptation (Pearlin et al., 2005). Accordingly, parenting an individual with DD may function as a contextual risk factor associated with an increased likelihood of chronic stress at the group level (Barker et al., 2014; Christopher et al., 2023; Dembo et al., 2025). At the same time, there is considerable heterogeneity in how parents experience and adapt to ongoing caregiving demands. A substantial body of research indicates that protective factors such as social support, adaptive coping strategies, and psychological resources, may buffer the adverse effects of sustained stress exposure (DaWalt et al., 2025; Montirosso et al., 2021; Rakap & Vural-Batik, 2024; Zaidman-Zait et al., 2017). Importantly, beyond these caregiving-related demands, parents of individuals with DD also encounter the same types of daily stressors experienced by the general population (Piazza et al., 2013).

### Daily stress

Daily stressors are relatively minor events or challenges, such as interpersonal conflicts or work deadlines (Almeida, 2005), that have been shown to impact daily well-being and health (Haight et al., 2023). Over time, the cumulative impact of these everyday stressors can contribute to long-term mental and physical health problems (Charles et al., 2013; Rush et al., 2025) and increased mortality risk (Jeong et al., 2016). According to the daily stress process model (Almeida, 2005, 2024), daily stress is conceptualized as an interconnected, multi-component process through which everyday stressors influence health and well-being. The model draws on stress and coping theories (Lazarus & Folkman, 1984; Pearlin et al., 2005) and is supported by extensive daily diary research linking everyday stressors to emotional, physiological, and health-related outcomes (Almeida, 2024).

The core components of the model capture both exposure to daily stressors and individuals’ interpretations and responses across affective, cognitive, behavioral, and neuroendocrine domains (Almeida, 2024; Smyth et al., 2023). Stressor *exposure* refers to the frequency of daily stressor occurrence, whereas stressor *appraisal* involves cognitive evaluations of a stressor’s severity and perceived threat or disruption to daily functioning. *Perceived control* reflects beliefs about one’s capacity to manage the situation or to influence outcomes. *Emotional experiences* refer to the affective responses associated with exposure to daily stressors, including both immediate emotional reactions (e.g., anger, sadness, anxiety) and changes in overall negative affect associated with the presence versus absence of stressors on a given day, commonly conceptualized as negative affective reactivity (Charles et al., 2013).

Although these components are conceptually interrelated and central to stress-related outcomes (Monroe, 2008), prior research has typically examined them in isolation or in limited combinations, most often focusing on stressor exposure and affective reactivity (e.g., Darling et al., 2025; Piazza et al., 2013). To our knowledge, no study has examined these components simultaneously as an integrated system. As a result, less is known about how they co-occur and are organized, particularly in the context of chronic stress.

Although repeated daily stressors may contribute to chronic stress, chronic stress represents a broader, ongoing contextual vulnerability that shapes individuals’ perceptions of and responses to everyday stressors. In turn, this heightened vulnerability may amplify both exposure to daily stressors and the intensity of emotional and physiological reactions to them (Almeida, 2005). Recent research indicates that cumulative chronic stress across multiple life domains (e.g., health, financial, relationship) can amplify the effects of daily stress exposure and impact physical and emotional health (Haight et al., 2023; Lockwood et al., 2022). Moreover, the accumulation of daily stressors may contribute to the worsening of chronic stress, while chronic stress may, in turn, heighten sensitivity to daily stressors, suggesting a reciprocal relationship in which each process reinforces the other (Crosswell et al., 2022; Lockwood et al., 2022).

Socioeconomic factors also contribute to these pathways. Daily diary studies indicate that sociodemographic factors such as educational attainment are associated with daily stressor exposure and severity, such that individuals with higher education may report more stressor exposure but tend to appraise them as less severe (Grzywacz et al., 2004; Surachman et al., 2019). Consistent with differential vulnerability models, socioeconomic status may also moderate the strength of associations between daily stressors and mental and physical health (Christopher et al., 2023; Grzywacz et al., 2004), suggesting that it may function as both a contextual risk and a buffering factor within daily stress processes.

The relationship between parents’ chronic caregiving stress and adverse health outcomes may partly be explained by how parents perceive and respond to daily stressors. Consistent with prior research on the intersection of daily and chronic stress, studies indicate that parents of children with DD experience not only a higher frequency of daily stressors but also greater negative affective reactivity to these stressors, compared to parents of children without DD (Barker et al., 2014; Seltzer et al., 2009). These findings suggest that daily stress experienced by parents of children with DD may be heightened within a broader context of ongoing caregiving demands and challenges, contributing to elevated cumulative burden relative to other parents. However, most studies have focused only on stressor exposure and affective reactivity, rather than providing a comprehensive analysis of the cognitive, emotional, and psychological components of daily stress under parenting caregiving conditions. A more nuanced understanding of how these components interconnect and linked to mental health outcomes using network analysis is warranted. To address this gap, we applied a network analytic framework to conceptualize the daily stress process as a system of interrelated components rather than as isolated predictors, consistent with the conceptualization of the daily stress process (Almeida, 2005).

### Network analysis

In network models, *nodes* represent specific variables, while *edges –* the lines connecting nodes – reflect conditional associations between pairs of variables (Epskamp et al., 2018). Network analysis offers a system-level statistical approach that can estimate and visualize the structure of complex systems of mutually influencing components composed of interdependent components (Borsboom, 2017). In addition, network models provide quantitative metrics at both the global and node levels (Epskamp & Fried, 2018).

At the global level, metrics such as connectivity and density reflect the overall strength of interconnections among components and provide insight into the network’s structural organization. A dense, more tightly connected network may signal reduced psychological flexibility and heightened vulnerability to external perturbations (Robinaugh et al., 2019). At the node level, centrality indicates the relative influence of individual nodes within the network.

Central nodes are more broadly connected to other nodes and may therefore hold structurally influential positions within the network system (Bringmann et al., 2019). Because of their structural position, central nodes may may serve as a potential strategic intervention target, as changes in highly connected symptoms may be associated with broader changes across the network through cascading effects (Lancee et al., 2023). For example, network analyses of an internet-based intervention for depression demonstrated that improvements in central symptoms (e.g., sadness and indecision) during treatment were associated with concurrent improvements in several other symptoms (e.g., fatigue, concentration difficulties), consistent with a cascading pattern of change (Mullarkey et al., 2020).

A node’s *predictability* quantifies the extent to which its variance explained by its directly connected nodes, conceptually similar to the proportion of variance explained in regression models (Epskamp & Fried, 2018).

### Present study

The present study applies network analysis to compare the structural organization and connectivity of daily stress process components between mothers of individuals with DD and a comparison group of mothers of children without DD. Network models estimate unique conditional associations among stress components simultaneously, without imposing directional assumptions. This approach allows us to examine how key components of the daily stress process, including stressor exposure, severity, risk appraisal, perceived control, and emotional responses, are organized and interrelated within each group. In addition to estimating these associations, network analysis provides a visual and quantitative representation of overall network topology, including node centrality and global connectivity (Epskamp & Fried, 2018).

Including subsequent depressive symptoms in the network also allows us to examine how daily stress components are connected to later psychological outcomes and to identify which components show the strongest unique associations with depressive symptoms. Importantly, this approach characterizes patterns of interdependence rather than directional or causal pathways (Huang et al., 2023).

Advancing understanding of how daily stress components are organized is important because these components represent proximal and potentially modifiable processes that may inform intervention aimed at improving mental health among individuals at heightened risk (Smyth et al., 2023). Specifically, the present study purposes were to (1) examine and visualize the structure and interconnections among daily stress process components in two samples: mothers of adolescents and adults with DD and mothers of adolescents and adults without DD; (2) Compare network topology and global connectivity between the groups to evaluate whether caregiving stress context is associated with differences in the structural organization of daily stress components (van Borkulo et al., 2023); and (3) investigate how daily stress components are structurally connected to subsequent depressive symptoms within each group’s daily stress components network.

## Method

### Participants

Participants were drawn from three ongoing parallel longitudinal studies that together formed two analytic cohorts: (1) Families of Adolescents and Adults with Autism (AAA; https://family.waisman.wisc.edu/autism/), (2) Families of Adolescents and Adults with Fragile X Syndrome (FXS; https://family.waisman.wisc.edu/fxs-fmr1-associated-conditions/), which were combined to form a single DD cohort, and (3) the *National Survey of Midlife in United States* (MIDUS) (https://www.icpsr.umich.edu/web/ICPSR/series/203), which was the source of data for the comparison cohort. All studies included an 8-day daily diary component comprised of consecutive daily telephone interviews asking participants to report on experiences of the past 24 hours. Approximately two years later (*M* = 22.80 months; SD = 8.10) participants provided an assessment of depressive symptoms.

#### Mothers of adolescents and adults with developmental disabilities (DD group)

In the ongoing AAA study, families met the following criteria: (a) included a child age 10 or older; (b) child received an independent diagnosis of autism spectrum disorder from a professional, as reported by parents; and (c) scores on the Autism Diagnostic Interview–Revised administered by research staff, were consistent with the parental diagnostic report. Data were collected across nine waves between 1998 and 2021 (Dembo et al., 2025; Hong et al., 2023). In the ongoing FXS study, mothers were required to be the biological parent of a son or daughter with FXS, age 12 or older. In addition, documentation from an appropriate health care professional confirming that the child had full mutation of the *FMR1* gene was required. Data were collected across six waves starting in 2008.

The present sample began with 267 mothers who had a child with DD who participated in the daily diary study (*n* = 132 from the AAA study, conducted at wave 5, 2006–2007; *n* = 135 from the FXS study, conducted at wave 1, 2006–2007). We excluded mothers who completed fewer than four days out of the eight-day dairy data collection (*n* = 3) and mothers who did not report any stressors over any of the eight days (*n* = 6), resulting in a sample of 258 in the DD group. The decision to combine the two groups is consistent with past research and enhances statistical power. Supporting this decision, there were no significant differences between mothers from the AAA and the FXS samples on any study variable (i.e., stressor exposure, severity, negative emotions, perceived control, risk appraisal, negative affective reactivity, and depressive symptoms at time 2) except for mothers’ age, where mothers in the FXS group were significantly younger than mothers in the AAA study (*p* < .001). (see Supplementary Materials, Tables S1 and S2).

#### Mothers of adolescents and adults without disabilities (MIDUS comparison group)

The comparison sample of mothers was drawn from participants in the MIDUS study, specifically from those who participated in both the Daily Diary and Biomarker projects at MIDUS 2 (2004–2009) (Ryff & Almeida, 2017), as well as in the core survey administered to all MIDUS participants. Consistent with past research, we excluded from the comparison group MIDUS mothers of children with any DDs, mental health conditions, or those who had experienced the death of a child. The data used to exclude these cases were obtained from the MIDUS 2 baseline survey, which is linked to both the Daily Diary and Biomarker projects. In addition, mothers from the initial eligible MIDUS sample of 343 who completed fewer than four days of the diary study (*n* = 8, 2.28%) or did not report any stressors (*n* = 15, 4.28%) were excluded. Accordingly, 320 MIDUS mothers were eligible for inclusion in the current study’s comparison group.

Given that the Network Comparison Test (NCT) is sensitive to differences in sample size between groups (van Borkulo et al., 2023), we selected a demographically comparable subsample from the eligible MIDUS participants to match the DD group in size (*n* = 258). Propensity scores were estimated using logistic regression with age, marital status, education, and household income as predictors (Rosenbaum & Rubin, 1984). Comparison participants were selected using a stratified propensity score matching procedure that combined quintile-based subclassification with exact matching on marital status to improve covariate balance Specifically, the propensity score distribution of the DD group was divided into five strata based on quintiles. Within each propensity score stratum and marital status category, comparison participants were randomly sampled to closely match the frequency of DD participants, yielding a matched sample of 258 mothers. Covariate balance was evaluated using absolute standardized mean differences (SMDs). All SMDs were below 0.20 after matching, indicating acceptable balance between groups, with most below 0.10 indicating acceptable balance between groups (Stuart, 2010).

As a robustness check, we conducted a sensitivity analysis using an alternative propensity score full matching approach based on the same covariates (Hansen, 2004). Results of the NCT were substantively unchanged, indicating that group differences in network structure and global strength were robust to the matching method (see Supplementary Materials, S6).

The DD and MIDUS comparison groups did not differ significantly in age (DD: *M* = 55.07, *SD* = 9.77; MIDUS: *M* = 53.98, *SD* = 10.68), marital status (77.5% vs. 72.1%), employment status, or number of children (all ps > .05; see Table S2). Most mothers identified as non-Hispanic White (95% and 94%, respectively). Approximately two thirds were employed (67.3% and 69.4%). Nearly half (46%) of mothers in the MIDUS group reported a co-resident child, whereas 71.7% of mothers in the DD group had a co-resident child with DD.

### Measures

#### Daily stress measures

Daily stressors were assessed using the daily inventory of stressor events (DISE), part of the Daily Diary Study (Almeida et al., 2002, 2025). For eight consecutive evenings, participants reported whether they had experienced specific stressors during the previous 24 hours (1 = yes, 0 = no), including arguments, avoided arguments, work- and home-related stressors, network stressors (events affecting close others), discrimination, or other unlisted stressors. For each endorsed stressor, participants completed follow-up questions assessing multiple aspects of the stressor experience. Specifically, mothers rated the perceived severity of the event (i.e., stressor severity; *“How stressful was this for you?*”) and their perceived control over the situation (i.e., perceived control; “*How much control did you have over the situation?*”). Risk appraisal was assessed by asking mothers to rate the degree to which the stressor posed a threat across six life domains: daily routines, others’ perceptions of them, financial well-being, future plans, personal health and safety, and self-concept (i.e., risk appraisal; “How much were the following things at risk in this situation?”).

Participants also reported the extent to which each stressor elicited negative emotions, including sadness, nervousness/anxiety, anger, and shame using the prompt “How much did you feel [the specific emotion]?”. All items were rated on a 4-point scale ranging from 0 = not at all to 3 = a lot (see Table 1 for descriptions and scoring). Follow-up questions assessing severity, appraisal, perceived control, and emotional reactions were administered only on days when a stressor was reported. Participants who did not report any stressors on any day during the diary period were excluded, as these components were assessed only on stressor days, resulting in structurally missing data for these individuals.

**Table 1.** Daily stress measures derived from the daily inventory of stressor events (DISE; Almeida et al., 2002)

Measure	Description	Response scale	Daily score	Aggregated score
Stressor exposure	Experience of specific daily stressors (i.e., arguments, argument avoidance, work, home, network stressors)	0 = no, 1 = yes	Sum of endorsed stressors per day	Mean score across all diary days
Stressor severity	Rated severity of each endorsed stressor: “How stressful was this for you?”	0 (not at all) to 4 (a lot)	Mean score across endorsed stressors per day	Mean score across all days with reported stressors
Perceived control	Rated perceived control over each stressor: “How much control did you have over the situation?”	0 (not at all) to 4 (a lot)	Mean score across endorsed stressors per day	Mean score across all days with reported stressors
Risk appraisals	Rated perceived risk across six domains: “How much were the following things at risk in this situation?”	0 (not at all) to 4 (a lot)	Mean score per domain, averaged across stressors	Mean score across all days with reported stressors
Negative Emotions	Emotional response per stressor: “How much did you feel [emotion]?” (sadness, anxiety, anger, shame)	0 (not at all) to 4 (a lot)	Mean score across emotions per stressor, averaged across stressors	Mean score across all days with reported stressors

*Note.* Higher levels of risk appraisal indicate appraising stressors as more threatening; Perceived control was reverse scored, so higher values indicate perception of less control.

#### Negative affective reactivity

Each day, participants rated how much they experienced 14 negative affective states (e.g., feeling restless, nervous, worthless, irritable, and ashamed) adapted from the Nonspecific Psychological Distress Scale and the PANAS (Kessler et al., 2002; Watson et al., 1988), on a 5-point scale ranging from 0 (*none of the time*) to 4 (*all of the time*). Items were averaged each day to form a daily negative affect score. Day-level internal consistency was high (Cronbach’s *α*: DD group sample = 0.92; MIDUS comparison group = 0.88).

Negative affective reactivity was measured by examining within-person changes in negative affect on days with versus without stressor exposure. Multilevel modeling was used to estimate affective reactivity. The model included random intercepts to represent each participant’s average level of negative affect across days, and random slopes to capture the extent to which negative affect differed on stressor days versus non-stressor days. A reactivity slope was extracted for each participant to represent their individual level of affective reactivity.

#### Depressive symptoms

Maternal depressive symptoms were measured using the 20-item *Center for Epidemiologic Studies Depression Scale* (CES-D) (Radloff, 1977). Mothers reported how many days in the past week they experienced each of the 20 depressive symptoms, using a scale ranging from 0 (*never*) to 3 (*5–7 days*). Total mean scores were calculated, with higher scores indicating higher levels of depressive symptoms (Cronbach’s *α*: DD group sample *α* = 0.92; MIDUS comparison group *α* = 0.84). Note that for the MIDUS participants, the CES-D was collected in a supplemental sample (i.e., the Biomarker component).

### Data analysis

In the current study, the term “daily stress network” refers to the multivariate network including key components of the daily stress process, including stressor exposure, severity, risk appraisal, perceived control, negative emotions, and negative affective reactivity. Because stressor-related components were assessed only on days when a stressor was reported, the number of stressor-day observations varied across participants. Accordingly, analyses were conducted using aggregated person-level estimates of these components averaged across all available stressor days. This approach is consistent with the study’s focus on between-person differences in the structure of daily stress components.

#### Network estimation and visualization

We estimated network structure of the daily stress process components for each group separately. Following current recommendations (Isvoranu & Epskamp, 2023), we estimated undirected regularized partial correlation networks using Gaussian Graphical Models (GGM) (Epskamp & Fried, 2018). In these undirected networks, edges represent partial statistical associations among components of daily stress process and do not imply directionality or causality. GGM applies the graphical least absolute shrinkage and selection operator as regularization technique, which reduces the risk of false positive edges and obtains parsimonious networks (Epskamp et al., 2018). We used the default tuning hyperparameter value (γ = 0.5) to ensure a conservative network estimation (Isvoranu & Epskamp, 2023). Analyses were conducted in RStudio (R version 4.3.0) using the R package bootnet (Epskamp et al., 2018). For network visualization, we used the qgraph package which uses the Fruchterman-Reingold algorithm to position strongly connected nodes closer together and more centrally within the network. Thicker edges indicate stronger connections between nodes, with blue edges representing positive associations and red edges representing negative associations. To facilitate visual comparison between groups’ networks, we constrained network layouts of the two groups to an average layout.

#### Network comparison

To examine differences between the DD group and the MIDUS comparison group networks, we used the *NetworkComparisonTest* (NCT; van Borkulo et al., 2023). The NCT is a permutation-based method in which group membership is repeatedly shuffled to generate an empirical null distribution of differences in network structure and connectivity. Observed group differences are then compared with this null distribution to determine statistical significance. During each permutation, participants are reassigned to groups while preserving the original sample sizes, and networks are re-estimated to determine whether the observed differences exceed those expected by chance. In the present study, 1,000 permutations were used to evaluate differences in network structure, global connectivity, and individual edge weights.

NCT examines differences in three aspects of network structure: (1) overall network structure, defined as differences in the distribution of edge weights; (2) overall network connectivity, which is operationalized as global strength (the total sum of absolute edge weights in each network); and (3) individual edge weights, which assess whether specific connections between nodes differ between groups. To assess the robustness of the group differences, we conducted sensitivity analyses to examine whether the observed differences in daily stress networks could be explained by sociodemographic characteristics. Specifically, prior to network estimation, daily stress components were adjusted for age, education, household income, and marital status by removing variance associated with these covariates. Networks were then re-estimated, and the NCT was conducted (see Supplementary Materials, S7).

#### Centrality and predictability

To examine the degree to which nodes are connected to other nodes in the network we examined strength centrality (i.e., the sum of edge weights of all edges connected to that node) (Haslbeck & Fried, 2017). Strength centrality has shown to be one of the most reliable centrality indices (Bringmann et al., 2019; Robinaugh et al., 2016). In addition, we estimated node predictability, which quantifies how well each node is predicted by all other nodes in the network that share a direct edge with it (Haslbeck & Waldorp, 2018). Predictability is plotted using circular pie-charts around the nodes. The colored areas around the nodes indicate the percentage of variance explained by the edges that are connected to that node.

#### Stability and accuracy

To determine the accuracy of the edge weights across the estimated networks we used the nonparametric bootstrap to generate bootstrapped confidence intervals around the edge weights. To examine the stability of the networks we computed the correlation stability coefficient (CS) for connection strengths. The CS represents the maximum proportion of respondents that can be dropped, such that with 95% probability the correlation between the connection strength of the original network and the connection strength of the networks based on subsets. A CS (cor = 0.70) > 0.50 is regarded indicates strong stability and interpretability implemented in R-package bootnet (Epskamp et al., 2018).

#### Daily stress and depressive symptoms network

To examine connecting pathways between components of the daily stress process to subsequent depressive symptoms, we estimated the network structure and the shortest path analysis using Dijkstra’s algorithm (Isvoranu et al., 2017) which identifies the most efficient connecting routes. These analyses were first conducted with the DD group and subsequently with the MIDUS comparison group. However, as only 115 MIDUS participants completed the CES-D after the daily diary period, these results are exploratory.

## Results

### Descriptive statistics

Table 2 presents descriptive statistics, bivariate correlations, and group differences (i.e., DD and MIDUS) for the study variables. Correlation patterns were largely consistent across groups, with moderate to strong associations among daily stressor severity, negative emotions, negative affective reactivity, and later depressive symptoms. Independent samples *t*-tests indicated that mothers in the DD group reported significantly higher levels of daily stressor exposure, severity, negative emotions, risk appraisals, negative affective reactivity, and depressive symptoms compared to the MIDUS comparison group (*p* < .05). Perceived control was the only variable that did not differ significantly between groups.

**Table 2.** Descriptive statistics of daily stress processes by group

	DD group	MIDUS group							
	Mean (SD)	Mean (SD)	*t*-Values	Stressor severity	Negative emotions	Perceived control	Risk appraisals	Affective reactivity	Depressive sym. T2
Stressor exposure	0.92 (0.64)	0.63 (0.41)	−6.25**	.14*	.17**	.05	.40**	.09	.19**
Stressor severity	2.05 (0.50)	1.84 (0.52)	−4.64**		.58**	.28**	.41**	.34**	.22**
Negative emotions	0.83 (0.38)	0.74 (0.41)	−2.70*			.20**	.46**	.57**	.35**
Perceived control	1.75 (0.69)	1.69 (0.83)	−0.95				.16**	.06	.12
Risk appraisals	0.42 (0.30)	0.35 (0.27)	−2.88*					.45**	.35**
Affective reactivity	0.16 (0.07)	0.14 (0.08)	−2.91*						.53**
Depressive sym. T2*	0.63 (52)	0.30 (0.28)	−7.89**						
**MIDUS group**									
Stressor exposure				.06	−.03	−.01	.15*	.04	.21*
Stressor severity					.55**	.09	.45**	.29**	.19*
Negative emotions						.03	.38**	.42**	.23*
Perceived control							−.05	−.01	0.11
Risk appraisals								.42**	.28**
Affective reactivity									.34**

*Note. n* = 258; except for MIDUS depressive symptoms (*n* = 115); *p < 0.01, **p < 0.001.

### Networks estimation and visualization

**Figure 1.**
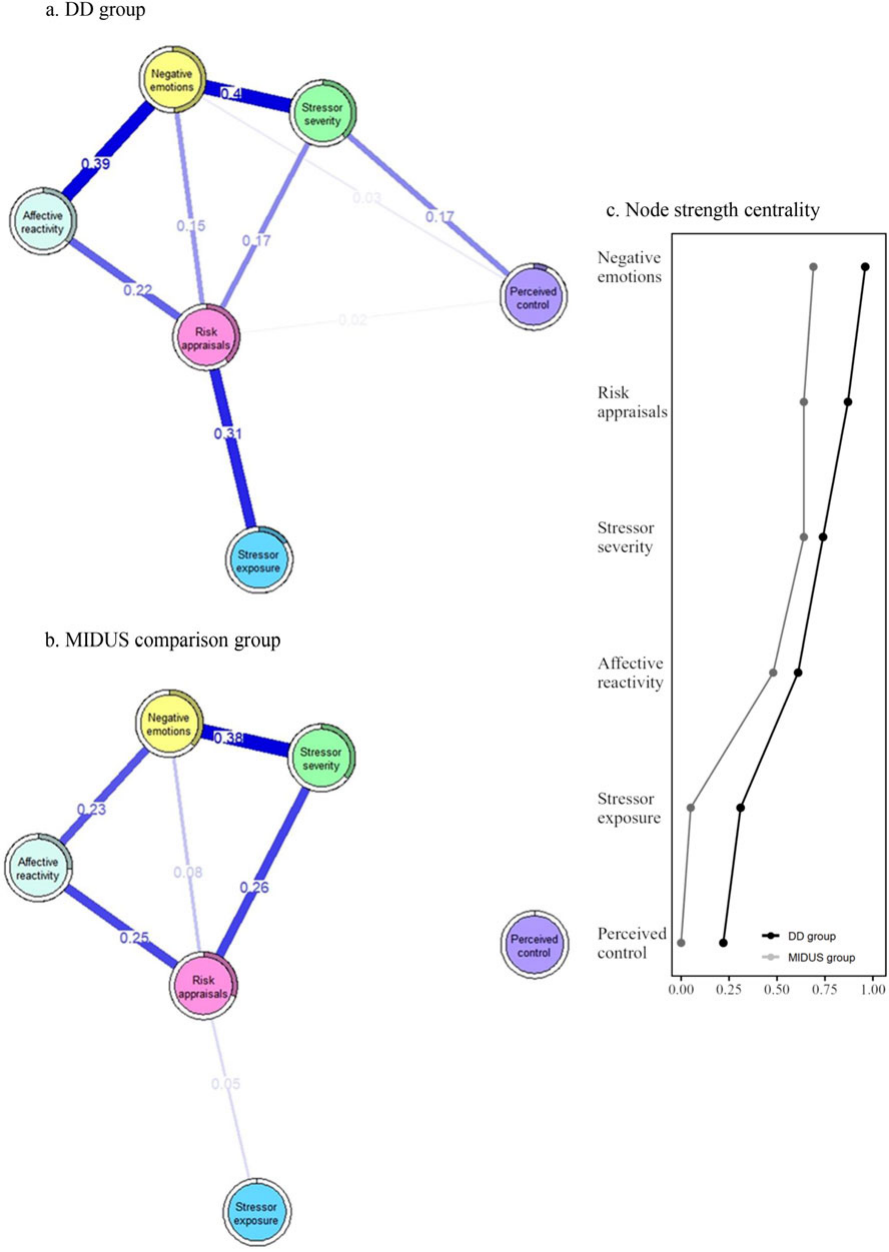
Regularized network graphs and node strength centrality of daily stress by group. (a) Network graph for the DD group; (b). Network graph for the MIDUS comparison group. In both panels (a). and (b). edge thickness represents the strength of associations, with blue edges indicating positive associations, and the colored segments of the rings around each node indicate the proportion of variance explained by all other nodes in the network. To ensure consistency, the same average layout was applied to both networks. (c). Node strength centrality of the daily stress networks for the DD and MIDUS comparison groups. The *x*-axis represents the *z*-standardized strength centrality values, and the *y*-axis indicates the daily stress processes.

Networks of daily stress process components for each group are visualized in Figure 1a. Visual inspection of the networks revealed that across both groups, consistent edges were observed between stressor severity, risk appraisal, negative emotions, and negative affective reactivity. However, edges between perceived control and severity, and between stressor exposure and risk appraisal, were observed only among mothers in the DD group (see Figure 1a). Strength centrality scores are displayed in Figure 1b. As shown in the figure, negative emotions, risk appraisal, and stressor severity had the highest strength centrality in both groups, suggesting that these nodes play a significant role in the overall structure of daily stress process component networks, regardless of group status.

Figure 1a also depicts node predictability estimates for each group’s network nodes (depicted by rings around each node). The average node predictability for the daily stress network was 31% in the DD group and 21% in the MIDUS comparison group. In both networks, negative emotions exhibited the highest level of node predictability (DD: 49%; MIDUS: 37%), indicating that negative emotions were strongly explained by other variables in the network. Conversely, perceived control had the lowest predictability (DD: 7%; MIDUS: 0%), suggesting that perceived control may be independent of other factors within the daily stress network.

### Network comparisons

NCT indicated significant differences in overall network structure, defined by the presence or absence of edges (*M* = 0.263, *p* < .001). In addition, the networks differed in global strength (*S* = 0.61, *p* < .001). Higher global strength indicates greater connectivity among daily stress components rather than higher mean levels of stress, consistent with a more tightly interconnected network structure. Specifically, the DD group showed higher global strength than the MIDUS comparison group (1.86 vs. 1.25). These findings suggest that the daily stress network is denser and more interconnected in the DD group, indicating greater interdependence among components of the daily stress process.

Examination of edge weight differences indicated that the connection between Exposure and Risk Appraisals was significantly stronger in the DD group network than in the MIDUS comparison group (*E* = 0.26, *p* < .01). In addition, the edge between Severity and Perceived Control was present only in the DD group (*E* = 0.17, *p* < .05). These findings suggest that, for mothers in the DD group, daily stressor exposure is more strongly associated with threat-related appraisals, and that perceptions of control may be more tightly linked to how severe stressors are perceived.

In terms of differences in nodes’ strength centrality across the networks, several nodes in the DD group network had significantly higher strength centrality than these nodes in the MIDUS group network. These were stressor exposure (*C* = 0.264, *p* = .01; DD = 0.314, MIDUS = 0.048), negative emotions (*C* = 0.268, *p* = .02; DD = 0.962, MIDUS = 0.69), and perceived control (*C* = 0.220, *p* = .01; DD = 0.220, MIDUS = 0.00). These findings suggest that, in the context of chronic caregiving stress, specific daily stress components play a more central role in the structure of the daily stress process network than in the comparison group. Results from the sociodemographic-adjusted networks were substantively similar to those from the primary analyses, indicating that group differences in network structure and global connectivity were not fully explained by participants’ age, education, household income, or marital status (see Supplementary Materials, S7).

### Daily stress and depressive symptoms: DD group

**Figure 2.**
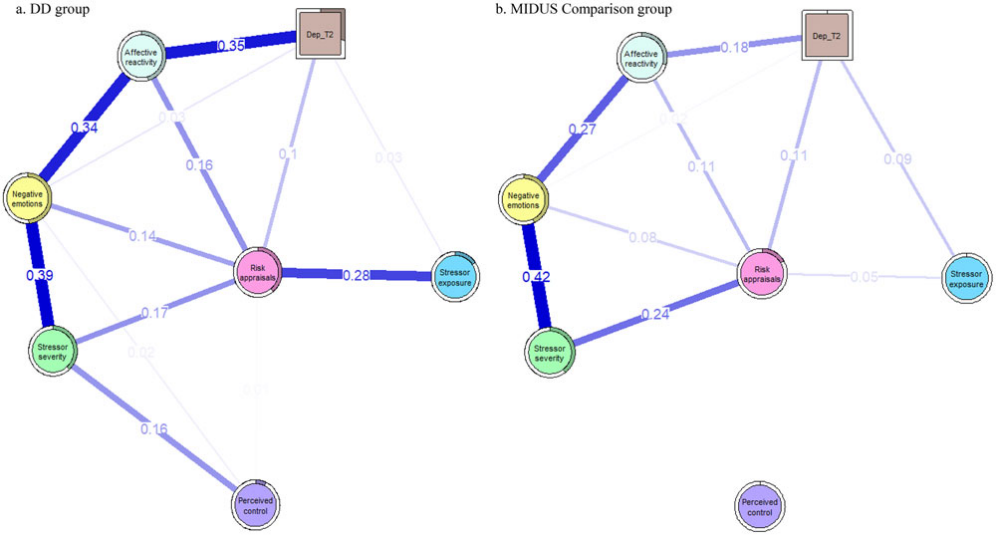
Figure 2 long description. Network graphs of daily stress process components and depressive symptoms at Time 2 by group. Edge thickness represents the strength of associations, with blue edges indicating positive associations. The colored segments of the rings around each node indicate the proportion of variance explained by all other nodes in the network.

Estimation of the network model specifically within the DD group (see Figure 2a) revealed that the predictability value for depressive symptoms was 29.4%, indicating that nearly one-third of the variance in depressive symptoms (measured on average nearly two years after daily stress measurement) was accounted by prior daily stress process components. Negative affective reactivity emerged as the most central stress process node, with the strongest direct connection with depressive symptoms, having the highest edge weight (.35). Risk appraisals were also directly linked with depressive symptoms, yet with a smaller edge weight (.11).

Findings from the shortest path analysis showed that negative affective reactivity was the only node directly connected to depressive symptoms (see Figure S1). All other pathways from daily stress components to depression were indirect and routed through negative affective reactivity as a linking node. The network framework allows for the simultaneous exploration of all direct and indirect relationships within a system of interrelated stress processes, revealing negative affective reactivity as a central mechanism to later depressive symptoms.

Specifically, stressor risk appraisals were linked to depression indirectly via negative affective reactivity, while other daily stressor nodes such as stressor severity and perceived control were connected to depressive symptoms through a longer sequence of nodes involving negative emotions and negative affective reactivity. These findings emphasize the central role of negative affective reactivity in the daily stress–depression pathway, highlighting its function as a potential bridge through which stress-related processes influence subsequent mental health outcomes among mothers of adolescents and adults with DD.

### MIDUS comparison group: daily stress and depressive symptoms

The exploratory network analysis for the MIDUS comparison group (Figure 2b) indicated overall similar paths linking components of daily stress process to depressive symptoms as observed in the DD group. Negative affective reactivity exhibited the strongest direct association with depressive symptoms (edge weight = 0.18), although this association was weaker than in the DD group. Similarly, the risk appraisals node was directly connected to depressive symptoms (edge weight = 0.11). Due to the relatively small and unequal sample sizes across groups, all between-group interpretations are descriptive and should be considered exploratory.

### Network stability

As shown in Figures S2–S9, the bootstrapped confidence intervals around the edge weights were relatively narrow, indicating adequate accuracy in the estimation of conditional associations in both networks. The centrality stability (CS) coefficient for strength centrality was CS = 0.67 for the DD group and CS = 0.59 for the MIDUS comparison group, both exceeding the recommended threshold of 0.50 for stable interpretation (Epskamp et al., 2018). For example, a CS value of 0.67 indicates that up to 67% of cases can be removed while retaining a high (≥.70) correlation between the original and subset strength centrality estimates. These results indicate that the centrality estimates are stable and can be interpreted with confidence.

## Discussion

The current study applied a network analysis approach to examine the structure of daily stress process components among mothers of individuals with DD compared with a matched sample of mothers of individuals without DD. Additionally, by including subsequent depressive symptoms within each group’s network, we examined how daily stress components were connected to later psychological outcomes. The present findings advance our understanding of daily stress in several important ways. First, the study conceptualizes daily stress as an interconnected system of cognitive and emotional components rather than a collection of separate variables (Borsboom, 2017; Robinaugh et al., 2019). Prior research has primarily focused on a limited subset of daily stress components, most often stressor exposure or negative affective reactivity (Piazza et al., 2013; Sin et al., 2015). In contrast, a network approach enables visualization and quantification of the complex interplay among multiple stress components and their associations with subsequent depressive symptoms.

Comparison of the two groups’ networks revealed differences in both overall structure (i.e., topology) and global connectivity. Mothers of individuals with DD exhibited a denser and more tightly interconnected network of daily stress, which may influence how daily stress contributes to depressive symptoms. Importantly, these differences remained significant after accounting for age, income, maternal education, and marital status, suggesting that they cannot be explained by sociodemographic factors.

A denser and more tightly interconnected network in the DD group might reflect reduced flexibility in how stress exposure and responses co-occur, potentially signaling heightened vulnerability to everyday stressors (Borsboom, 2017; Robinaugh et al., 2019). Such a pattern may, in turn, influence how daily stress contributes to depressive symptoms. These findings are consistent with the theoretical perspectives on daily stress suggesting that persistent parenting caregiving demands may function as an individual-level vulnerability factor that amplifies both the frequency and perceived severity of daily stressors, as well as emotional and physiological responses (Almeida, 2005).

Overall, these daily stress networks underscore the substantial and ongoing impact of parenting caregiving related stress on mothers of individuals with DD (Masefield et al., 2020), highlighting the cumulative toll of chronic stress on both physical and mental health, as well as the wear-and-tear associated with parenting a child with a DD (Dembo et al., 2023, 2025; Lockwood et al., 2022). These findings are consistent with prior research showing that indicators of chronic stress are associated with greater daily stressor exposure and heightened negative affective reactivity (Haight et al., 2023; Lockwood et al., 2022; Serido et al., 2004).

However, because chronic stress was not directly measured, these findings should be interpreted as evidence of structural differences between groups rather than causal evidence that chronic stress reorganizes daily stress processes. Future studies that directly assess chronic stress, particularly using longer intensive longitudinal designs, would allow researchers to examine how persistent stress conditions shape the structure of daily stress networks and influence later health outcomes while accounting for temporal order and directionality.

Beyond global differences, specific edge weights also differed across groups. Most notably, the heightened tendency of the DD group to interpret daily stressors as threatening may reflect the dual burden of chronic caregiving stress and more frequent exposure to daily stressors (Smith et al., 2010). Chronic caregiving stress may modify psychological mechanisms such as attentional biases or cognitive appraisal styles, leading individuals to appraise relatively minor daily experiences as more severe (McEwen, 1998). However, because network edges represent conditional associations rather than directional relationships, the present findings cannot determine the direction of these associations. Future longitudinal research is needed to clarify the temporal ordering of these associations.

Stressor risk appraisals emerged as a central node with the highest overall influence within the daily stress networks across both groups (Robinaugh et al., 2016). This stress component was connected to nearly all other nodes functioning as a key hub within the network, that links stressor exposure with other cognitive and emotional stress components. This finding aligns with appraisal theory, which emphasizes the significant role of cognitive evaluations in shaping stress responses (Lazarus, 1999). Recent research documenting robust associations between stressor appraisals and negative affect further supports the importance of appraisals in organizing stress-related experiences (Graham et al., 2025).

Extending prior research (Charles et al., 2013; Parrish et al., 2011; Wichers et al., 2009), emotional responses also emerged as a highly interconnected node within the network, and negative affective reactivity was closely associated with subsequent depressive symptoms, particularly among mothers in the DD group. Negative affective reactivity appeared to function as a bridging component linking cognitive appraisals, perceived control, and stressor exposure to later psychological outcomes. Together, these findings suggest that heightened emotional reactions to everyday stressors may represent a central organizing feature of the daily stress system. Among mothers of individuals with DD, stronger connections between negative affective reactivity and other stress components may reflect a more tightly coupled emotional response system in which daily stress experiences are more strongly interconnected and potentially less adaptive. Importantly, these associations reflect structural relationships within the stress network rather than causal pathways.

Components such as stressor risk appraisal and negative affective reactivity emerged as central nodes, suggesting that they occupy influential positions within the daily stress network. From a network perspective, highly connected components may function as leverage points, as changes in these components may be associated with broader shifts in related stress processes. Emerging network research further suggests that targeting highly central nodes may be associated with cascading effects across the broader network (James et al., 2024; Mullarkey et al., 2020; Strauss et al., 2020).

In addition to their central position within the network, prior research indicates that these components are modifiable. Intervention studies have shown that negative affective reactivity and cognitive and contextual aspects of stressor appraisal can be modified, promoting more adaptive responses to daily stressors (Castro et al., 2023; Leger et al., 2022; McIntyre et al., 2019). However, recent methodological work cautions that node centrality does not necessarily correspond to optimal intervention targets, particularly when centrality is based on cross-sectional networks (Van de Leemput et al., 2024). Accordingly, central nodes should be interpreted as highlighting potentially important processes within the stress system rather than definitive intervention targets. Future translational research should examine whether interventions such as emotion regulation training (Moradikia et al., 2016), acceptance-based interventions (Byrne et al., 2021), or mindfulness-based stress reduction (Bazzano et al., 2015) can modify these central stress components and, in turn, weaken the links between daily stress and depressive symptoms.

## Strengths, limitations & future research

To our knowledge, this is the first study to apply a network analytic framework to examine the structure of daily stress process components, their interrelations, and their associations with subsequent depressive symptoms among mothers of adolescents or adults with DD, compared with a matched sample of mothers of individuals without DD. The findings provide insight into the organization of daily stress components within this well-established stressful caregiving context, revealing that the DD group demonstrated a denser and more tightly interconnected network structure. Stressor risk appraisal and negative emotional responses, and affective reactivity emerged as central nodes within the network. In addition, structural links between daily stress components and depressive symptoms were stronger in the DD group. Given the high prevalence of DD, which affects approximately 1 in 6 children in the United States (Zablotsky et al., 2019), these results are relevant for a substantial number of families.

Despite the strengths of this study, several limitations should be acknowledged. First, although categorizing mothers based on caregiving context is grounded in extensive empirical evidence (da Estrela et al., 2018; Kong et al., 2025), this approach may obscure meaningful within-group variability. Contextual and child-related characteristics known to shape parents’ stress experiences (Barker et al., 2014; Montirosso et al., 2021; Rakap & Vural-Batik, 2024) may also influence how parents respond to daily stressors and the structure of daily stress networks. In addition, socioeconomic context may influence stress responses in complex ways and moderate associations among daily stress components (e.g., Christopher et al., 2023). The chronic nature of caregiving-related stress may also have implications for longer-term biological processes associated with aging and health. A growing body of research suggests that ongoing exposure to stress is associated with accelerated biological aging, including shorter telomere length, a marker of cellular aging (Brown et al., 2023; Lin & Epel, 2022).

Future research would benefit from incorporating additional indicators of stress and adaptation into network models, including biological and physiological measures (e.g., cortisol levels, cardiovascular reactivity), as well as contextual and psychological risk and protective factors. Integrating these indicators may help clarify mechanisms underlying parents’ caregiving stress and identify protective pathways that inform targeted interventions. Furthermore, although the current sample provides valuable insight into caregiving-related stress among mothers of individuals with DD, future studies should examine whether similar network patterns emerge in other chronically stressed populations, such as caregivers of individuals with chronic illnesses or those experiencing socioeconomic adversity.

Second, the relatively short daily diary period required aggregating the daily data for network modeling, which in turn prevented examination of within-person temporal dynamics among the stress components (Bringmann et al., 2013). Future research using longer periods of daily data collection would allow more nuanced understanding of how stressor exposure, cognitive appraisals, and emotional responses unfold over time and contribute to mental health outcomes.

An additional consideration is the potential influence of response styles in self-reported measures, which may inflate associations among variables and contribute to denser network structures (Fried & Cramer, 2017; Podsakoff et al., 2003). In the present study, however, networks were estimated using aggregated person-level components, such that edges represent unique conditional associations at the between-person level rather than variability-based indices. Furthermore, extensive daily diary research consistently demonstrates substantial within-person variability in stress and affect across days, suggesting that participants’ responses are sensitive to daily contexts rather than driven solely by stable reporting tendencies (Rush et al., 2019; Sliwinski et al., 2009). Thus, although response styles may contribute to shared variance among variables, the observed group differences in network density are unlikely to be fully explained by reporting tendencies and may instead reflect meaningful differences in the organization of daily stress components. Future research should examine potential reporting biases more directly using psychometric approaches (Naragon-Gainey & Stanton, 2025).

Finally, the study sample was predominantly non-Hispanic White and included limited representation of families with lower levels of education and income. Socioeconomic conditions may shape daily stress experiences (Surachman et al., 2019), and parents caregiving stress processes may differ across socioeconomic contexts due to differences in access to services, financial strain, and available social resources (Christopher et al., 2023; Hickey et al., 2021; Kim et al., 2020; Rivera-Figueroa et al., 2025). These considerations highlight the complexity of parental coping and adaptation across sociocultural contexts. Accordingly, the present findings should be interpreted given the sample’s demographic characteristics. Future research should examine whether the structure of daily stress networks generalizes to more socioeconomically and racially diverse populations.

Taken together, findings suggest that daily stress is best understood as a system of interrelated cognitive and emotional processes rather than as a set of independent components. Mothers of individuals with DD exhibited a more tightly interconnected network of daily stress components, suggesting that chronic caregiving demands may strengthen the links among components of daily stress process. This tighter coupling may, in turn, increase susceptibility to subsequent depressive symptoms. From an applied standpoint, results suggest that interventions may be most effective when they address multiple, highly interconnected components of the daily stress process. For example, this may involve reducing exposure to stressors, modifying stressor appraisals, and strengthening emotion regulation skills. Such approaches may help mitigate the cumulative impact of ongoing and daily stressors and support well-being among parents of individuals with DD.

## Supporting information

10.1017/S095457942610162X.sm001 Zaidman-Zait et al. supplementary material Zaidman-Zait et al. supplementary material

## Data Availability

De-identified MIDUS data from this study are free and publicly available. Information about accessing the data can be found on the MIDUS website: https://midus.wisc.edu/. The DD dataset analyzed in this study is not publicly available per IRB Due to the sensitive nature of the study, participants were assured that raw data would not be shared. Analytic code used to conduct the analyses presented in this study are available by emailing the corresponding author.

## Supplementary material

The supplementary material for this article can be found at https://doi.org/10.1017/S095457942610162X.

## Pre-registration statement

This study was not formally registered. Analyses were conducted using existing longitudinal datasets collected prior to the development of the present research questions. In addition, the study involved exploratory network analyses based on harmonized longitudinal datasets collected for other purposes. All study procedures, measures, and analytic decisions are reported transparently.

## Figure 2. Long description

A network graph comparing daily stress process components and depressive symptoms at Time 2 between two groups. Panel A: DD group. Panel B: MIDUS Comparison group. Each panel includes nodes representing different components: Affective reactivity, Negative emotions, Risk appraisal, Stress severity, Perceived control, Stress exposure, and Dep T2. Edges between nodes indicate associations, with edge thickness representing the strength of these associations. Blue edges indicate positive associations. The colored segments of the rings around each node show the proportion of variance explained by all other nodes in the network. In the DD group, notable associations include strong connections between Negative emotions and both Affective reactivity and Stress severity, and between Affective reactivity and Dep T2. In the MIDUS Comparison group, Negative emotions show strong associations with both Affective reactivity and Stress severity, but the connections to Dep T2 are weaker compared to the DD group.

Navigate back to Figure 2..
