# Supplementary material for: The network structure of daily stress process components: Comparing mothers of children with and without developmental disabilities
Source: Dev Psychopathol. 2026 Jul 7:1–13. Online ahead of print. doi: 10.1017/S095457942610162X (PMC13423608; doi:10.1017/S095457942610162X)
Supplement: Zaidman-Zait et al. supplementary material [file S095457942610162Xsup001.docx]

**Supplementary Materials**

**Supplementary S1. Demographic information**

[**Table S1.**](#TableS1) Descriptive statistics of daily stress processes for AAA & FXS subgroups

**Table S2.** Participant characteristics by group.

**Supplementary S2. Shortest path analysis**

**Figure S1.** Shortest path analysis between daily stress and later depressive symptoms

**Supplementary S3. analyses of edge-weights**

**Figure S2.** Nonparametric bootstrapped confidence intervals of estimated edge-weights DD-group

**Figure S3.** Nonparametric bootstrapped confidence intervals of estimated edge-weights MIDUS comparison group

**Figure S4.** Nonparametric bootstrapped difference tests (α = 0.05) of edge-weights (non-zero) DD-group

**Figure S5.** Nonparametric bootstrapped difference tests (α = 0.05) of edge-weights (non-zero) MIDUS comparison group

**Supplementary S4. Supplementary analyses of node centrality**

**Figure S6.** Case-dropping bootstrapped stability of the order of node strength DD-group

**Figure S7.** Case-dropping bootstrapped stability of the order of node strength MIDUS comparison group

**Figure S8.** Nonparametric bootstrapped difference tests (α = 0.05) of node strength DD-group

**Figure S9.** Nonparametric bootstrapped difference tests (α = 0.05) of node strength MIDUS comparison group

**Supplementary S5. Topological overlap between network nodes**

**Supplementary S6. Sensitivity analysis of matching procedure using full matching**

**Supplementary S7: Sociodemographic-Adjusted Networks**

**Supplementary S1. Demographic information**

Table S1. Descriptive statistics of daily stress processes for AAA & FXS subgroups

| Daily stressor processes | AAA group (n=127)  Mean (SD) | FXS group (n=131)  Mean (SD) | t-values |
| --- | --- | --- | --- |
| Stressor exposure | 0.96 (0.68) | 0.88 (0.45) | 1.09 |
| Stressor severity | 2.06 (0.490) | 2.03 (0.50) | 0.55 |
| Negative emotions | 0.83 (0.37) | 0.83 (0.38) | -0.12 |
| Perceived control | 1.75 (0.70) | 1.675 (0.68) | 0.00 |
| Risk appraisals | 0.41 (0.31) | 0.43 (0.30) | -0.47 |
| Affective reactivity | 0.17 (0.07) | 0.16 (0.07) | 0.27 |
| Depressive sym. T2 | 0.66 (0.55) | 0.60 (0.49) | -0.82 |

**p* < 0.05

Table S2. Participant characteristics by group.

|  | DD group | MIDUS comparison |  |
| --- | --- | --- | --- |
|  | M (SD) | M (SD) | t-values |
| Mothers' characteristics |  |  |  |
| Age (years) | 55.07 (9.77) | 53.98 (10.68) | -1.20 |
| Education level | 4.67 (1.78) | 4.4 (1.72) | -1.72 |
| Married | 77.50% | 72.10% | 1.42 |
| Race (non-Hispanic white = 1) | 95% | 94% |  |
| Employment status | 0.67 (0.47) | 0.69 (0.46) | .50 |
| Employed | 67.3% | 69.4% |  |
| Number of children | 2.70 (1.31) | 2.65 (1.27) | -.43 |
| Co-resident children (any child) |  | 46% |  |
| Co-resident children (child with DD) | 71.7% | - |  |

**p* < 0.05

**Supplementary S2. Shortest path analysis**


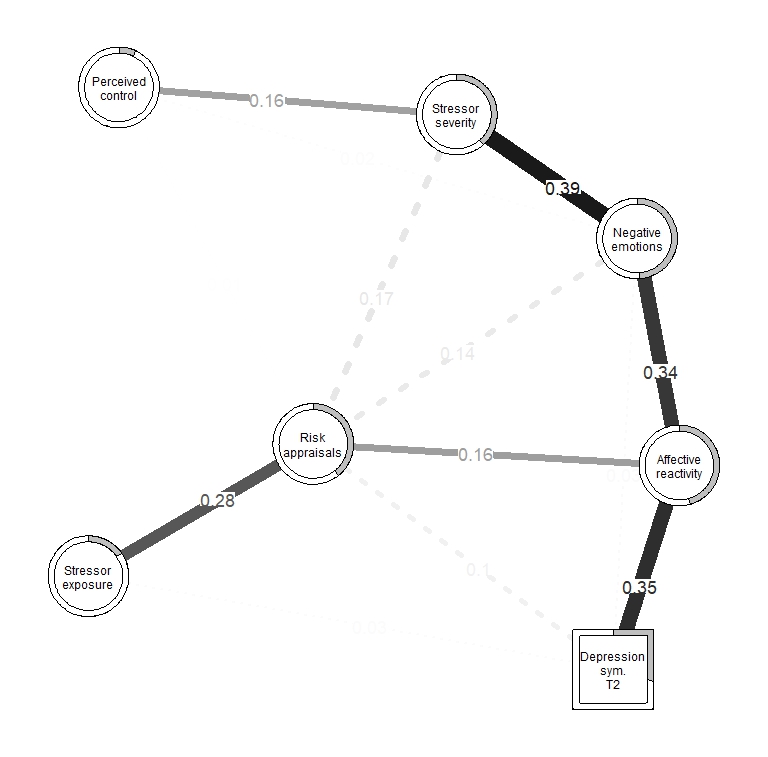


Figure S1. Network depicting the shortest paths between each daily stress components and depressive symptoms at T2 among mothers in the DD group. Bold solid edges indicate connections within the network that lie on at least one shortest path between daily stress components and depressive symptoms. Dashed lines represent conditional connections that do not lie on the shortest paths. Edge thickness reflects the strength of conditional associations. The wider the line, the stronger the correlation

**Supplementary S3. analyses of edge-weights**

To investigate the accuracy of edge weights estimation, bootstrapped confidence intervals (Figure S1 DD group & Figure S2 MIDUS comparison group) and bootstrapped difference tests (Figure S3 DD group & Figure S4 MIDUS comparison group) of edge weights were generated using nonparametric bootstrapping. This involved resampling the observations in the data with replacement over 2000 iterations.


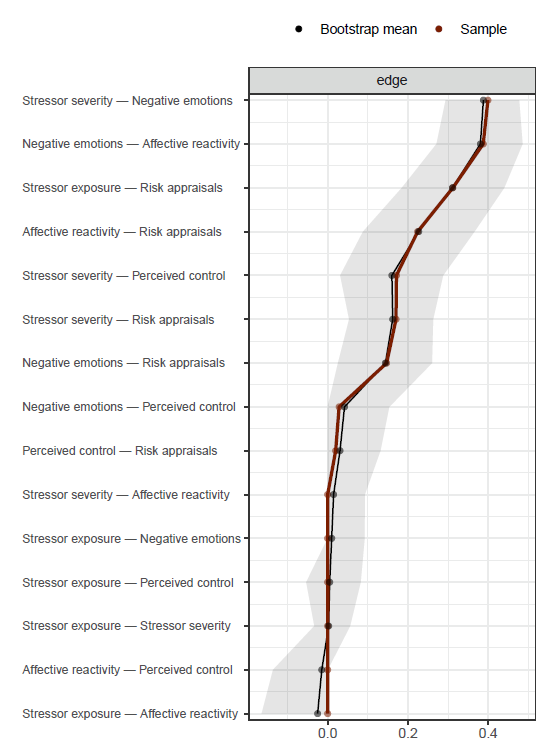


**Figure S2.** Nonparametric bootstrapped confidence intervals of estimated edge-weights for daily stress network model of the DD Group. Each horizontal line indicates one edge of the network. The red line depicts the sample values, the grey bar the 95% bootstrapped CIs and the black line the bootstrap mean. All bootstrap CIs were small enough to give a fair amount of confidence as to their stability.


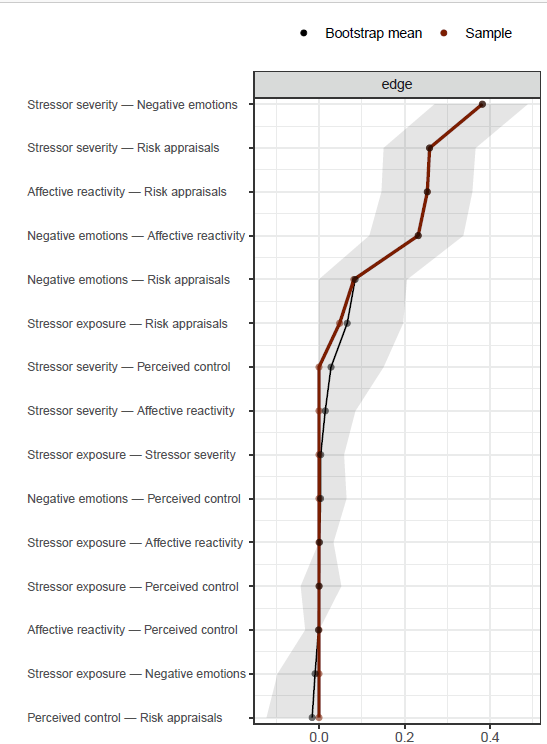


**Figure S3.** Nonparametric bootstrapped confidence intervals of estimated edge-weights for daily stress network model of the MIDUS comparison group. Each horizontal line indicates one edge of the network. The red line depicts the sample values, the grey bar the 95% bootstrapped CIs and the black line the bootstrap mean. All bootstrap CIs were small enough to give a fair amount of confidence as to their stability.

**
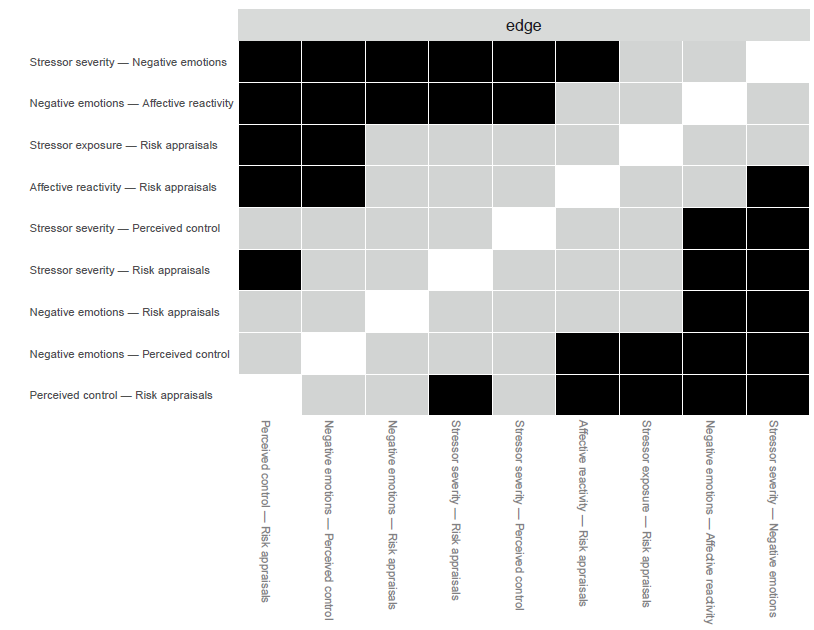
**

**Figure S4.** Nonparametric bootstrapped difference tests (α = 0.05) of edge-weights that were non-zero in the estimated daily stress network for the DD group. Black boxes indicate edges that differ significantly from one another, whereas gray boxes represent edges that do not differ significantly from one another.

**
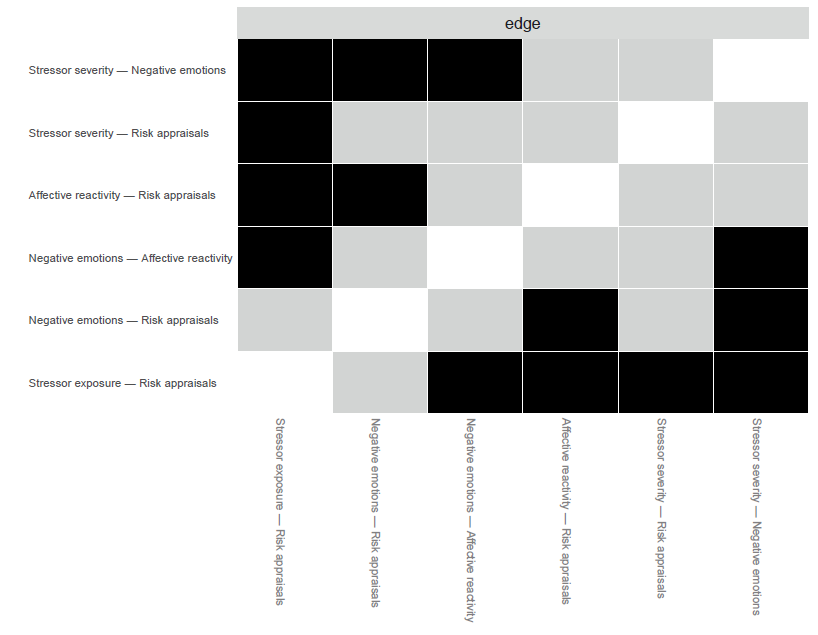
**

**Figure S5.** Nonparametric bootstrapped difference tests (α = 0.05) of edge-weights that were non-zero in the estimated daily stress network for the MIDUS comparison group. Black boxes indicate edges that differ significantly from one another, whereas gray boxes represent edges that do not differ significantly from one another.

**Supplementary S4. Supplementary analyses of node centrality**

The case-dropping for strength centrality in both groups' networks (Figure S5 for the DD group and Figure S6 for the MIDUS comparison group) indicates sufficient stability.

To examine whether the centrality of a given node was significantly different from other nodes nonparametric bootstrapped difference tests of strength centrality were conducted. The results for the DD group are presented in Figure A7, and for the MIDUS comparison group, the results are presented in Figure S8.


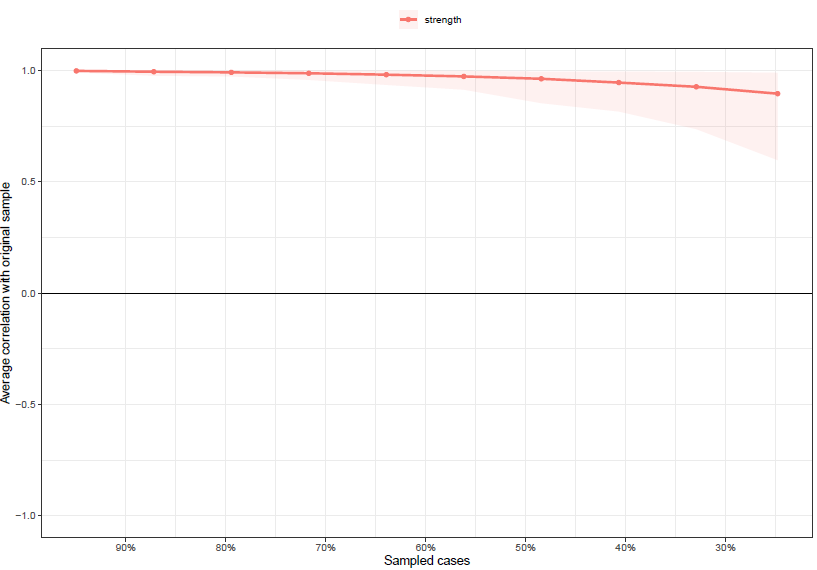


**Figure S6.**  Average correlations between the strength centrality of networks sampled by case-dropping subset bootstrap and the original sample (DD group)


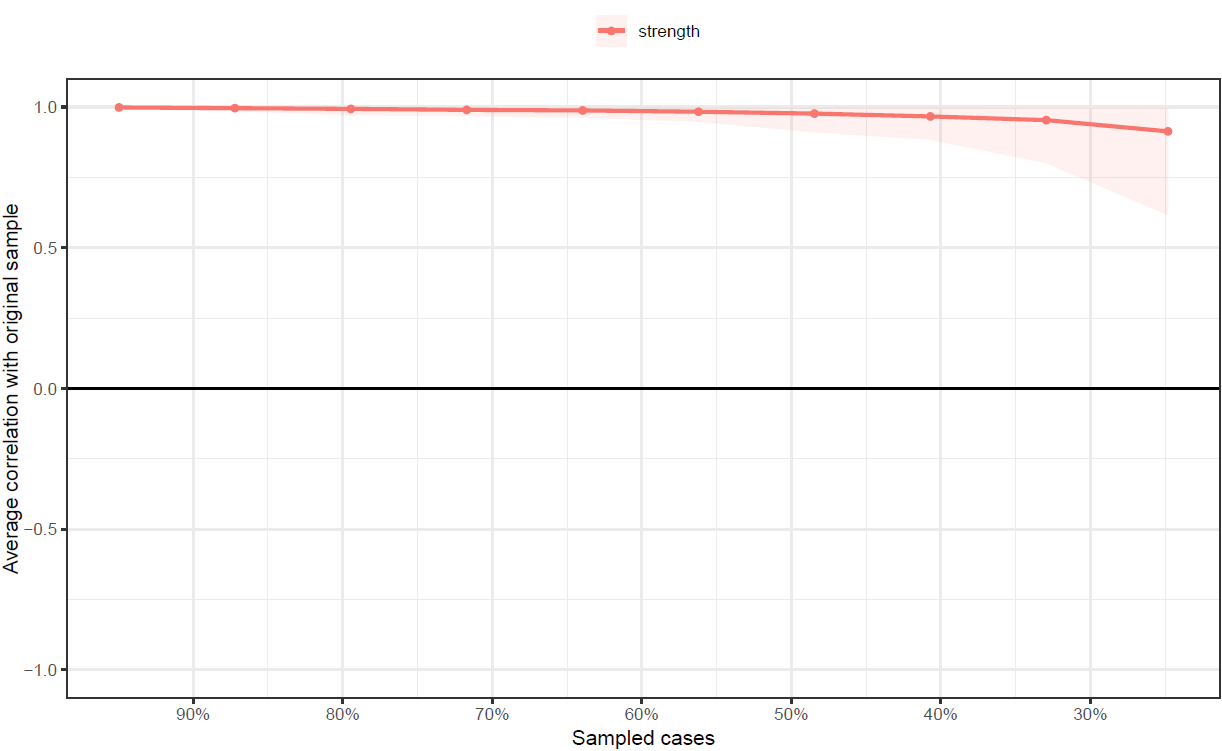


**Figure S7.** Average correlations between the strength centrality of networks sampled by case-dropping subset bootstrap and the original sample (MIDUS comparison group).


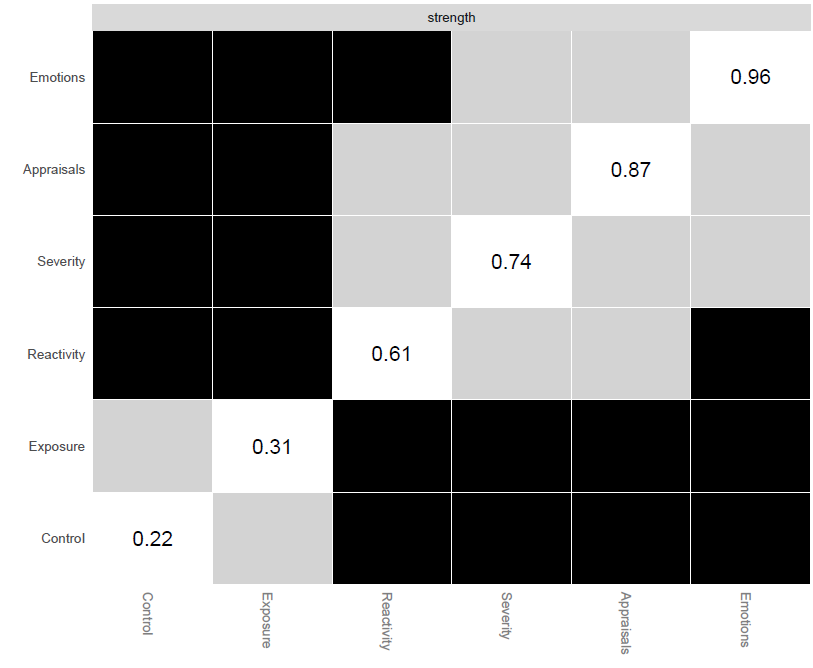


**Figure S8.** Nonparametric bootstrapped difference tests (α = 0.05) between node strength values (DD group): The figure represents nodes that differ significantly from one another, with gray boxes indicating nodes that do not differ significantly. The diagonal represents the standardized node strength.

*
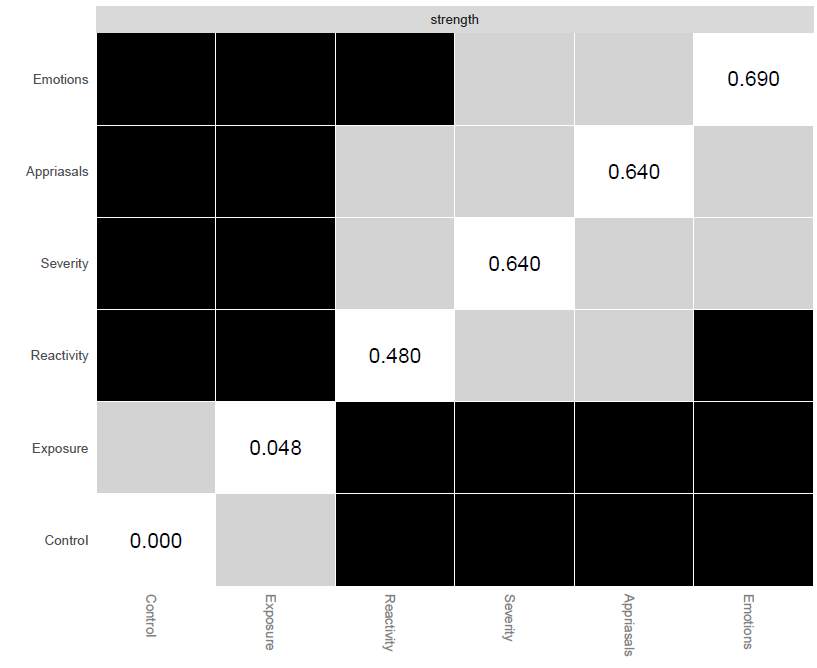
*

**Figure S9.** Nonparametric bootstrapped difference tests (α = 0.05) between node strength values (MIDUS comparison group): The figure represents nodes that differ significantly from one another, with gray boxes indicating nodes that do not differ significantly. The diagonal represents the standardized node strength.

**Supplementary S5. Topological overlap between network nodes**
 To assess topological overlap (i.e., node redundancy) between all pairs of nodes, we applied the *goldbricker* procedure. This procedure evaluates whether pairs of nodes exhibit statistically indistinguishable correlation patterns with all other nodes in the network. For the analyses, we used the goldbricker function from the *networktools* package in R (Jones, 2020). Following standard recommendations, node pairs were considered potentially redundant if fewer than 25% of their correlations with other nodes differed significantly (α = .05). A minimum correlation threshold of 0.50 was also applied. No nodes were identified as redundant in either the DD or MIDUS comparison networks, indicating that each daily stress component represented a distinct element within the network, including stressor-level negative emotions and negative affective reactivity.

**Supplementary S6.** **Sensitivity analysis of matching procedure using full matching**

Propensity scores were estimated using the same logistic regression specification as in the primary matching procedure, with group membership regressed on age, household income, years of education, and marital status. Full matching was implemented using the *MatchIt* package (Ho et al., 2011) using the “full” matching method with the DD group served as the reference group. Under this specification, the matching procedure is optimized to compare the DD group to a demographically similar non-DD group of mothers. The matching procedure groups participants in a way that minimizes differences between them on the characteristics used to create the propensity scores (Hansen, 2004). To ensure equal sample sizes (n = 258 per group), comparison participants were ranked by their full-matching weights, and the 258 participants with the highest weights were selected. Covariate balance was evaluated using absolute standardized mean differences (SMDs), all covariates had SMD below .10 indicating good balanced after full matching (Stuart, 2010).

Results of the Network Comparison Test using the full-matching derived sample were substantively identical to those obtained in the primary analysis. Specifically, NCT indicated significant differences in overall network structure (*M* = 0.312, *p* < .001). In addition, the DD group exhibited greater global network strength which reflects the overall level of connectivity among network components compared to the matched comparison group (1.86 vs. 1.12 respectively; *S* = 0.739, *p* < .001). In addition, the pattern of central nodes was consistent across matching approaches, with stressor risk appraisal and negative affective reactivity remaining among the most central components. Together, these findings indicate that the observed group differences are robust to the choice of matching method.

**Supplementary S7: Sociodemographic-Adjusted Networks**

To examine whether group differences in daily stress networks could be explained by sociodemographic characteristics, we conducted sensitivity analyses. Daily stress components were adjusted for age, education, household income, and marital status prior to network estimation. Networks were then re-estimated, and the NCT was conducted to evaluate differences in global strength and network structure. Results from the sociodemographic-adjusted networks were substantively identical to those obtained in the unadjusted networks. The NCT test of network structure invariance remained significant (M = 0.312, *p* = .003), indicating persistent differences in network structure between groups. In addition, the DD group continued to exhibit greater global network strength than the MIDUS comparison group (1.89 vs. 1.18, respectively; S = 0.707, *p* = .003), indicating greater interconnectivity among daily stress components. Overall, these findings indicate that the observed group differences are robust to sociodemographic adjustment.

**References**

Hansen, B. B. (2004). Full matching in an observational study of coaching for the SAT. *Journal of the American Statistical Association*, *99*(467), 609–618. https://doi.org/10.1198/016214504000000647

Ho, D. E., Imai, K., King, G., & Stuart, E. A. (2011). MatchIt : Nonparametric preprocessing for parametric causal inference. *Journal of Statistical Software*, *42*(8), 1–28. https://doi.org/10.18637/jss.v042.i08

Jones, P. J. (2020). Networktools: Tools for Identifying Important Nodes in Networks. R package version 1.2.3. *R*, *April 2017*. https://cran.r-project.org/package=networktools

Stuart, E. A. (2010). Matching methods for causal inference: A review and a look forward. *Statistical Science : A Review Journal of the Institute of Mathematical Statistics*, *25*(1), 1–21. https://doi.org/10.1214/09-STS313
